# Potential factors influencing lymphatic filariasis transmission in “hotspot” and “control” areas in Ghana: the importance of vectors

**DOI:** 10.1186/s40249-019-0520-1

**Published:** 2019-02-05

**Authors:** Sellase Pi-Bansa, Joseph Harold Nyarko Osei, Kwadwo Kyeremeh Frempong, Elizabeth Elhassan, Osei Kweku Akuoko, David Agyemang, Collins Ahorlu, Maxwell Alexander Appawu, Benjamin Guibehi Koudou, Michael David Wilson, Dziedzom Komi de Souza, Samuel Kweku Dadzie, Jürg Utzinger, Daniel Adjei Boakye

**Affiliations:** 1 Swiss Tropical and Public Health Institute, Basel, Switzerland; 2 University of Basel, Basel, Switzerland; 3 Noguchi Memorial Institute for Medical Research, College of Health Sciences, University of Ghana, Legon, Ghana; 4 Department of Animal Biology and Conservation Science, University of Ghana, Legon, Ghana; 5 SightSavers International, Ghana Office, Accra, Ghana; 6 African Regional Postgraduate Programme in Insect Science, University of Ghana, Legon, Ghana; 7 Vector Biology Department, Liverpool School of Tropical Medicine, Liverpool, UK; 8 Centre Suisse de Recherches Scientifiques en Côte d’Ivoire, Abidjan, Côte d’Ivoire

**Keywords:** Ghana, Hotspots, Lymphatic filariasis, Mass drug administration, Microfilariae, Systematic non-compliance, Vector control

## Abstract

**Background:**

Mass drug administration (MDA) programmes for the control of lymphatic filariasis in Ghana, have been ongoing in some endemic districts for 16 years. The current study aimed to assess factors that govern the success of MDA programmes for breaking transmission of lymphatic filariasis in Ghana.

**Methods:**

The study was undertaken in two “hotspot” districts (Ahanta West and Kassena Nankana West) and two control districts (Mpohor and Bongo) in Ghana. Mosquitoes were collected and identified using morphological and molecular tools. A proportion of the cibarial armatures of each species was examined. Dissections were performed on *Anopheles gambiae* for filarial worm detection. A questionnaire was administered to obtain information on MDA compliance and vector control activities. Data were compared between districts to determine factors that might explain persistent transmission of lymphatic filariasis.

**Results:**

High numbers of mosquitoes were sampled in Ahanta West district compared to Mpohor district (*F* = 16.09, *P* = 0.002). There was no significant difference between the numbers of mosquitoes collected in Kassena Nankana West and Bongo districts (*F* = 2.16, *P* = 0.185). *Mansonia* species were predominant in Ahanta West district. *An. coluzzii* mosquitoes were prevalent in all districts. *An. melas* with infected and infective filarial worms was found only in Ahanta West district. No differences were found in cibarial teeth numbers and shape for mosquito species in the surveyed districts. Reported MDA coverage was high in all districts. The average use of bednet and indoor residual spraying was 82.4 and 66.2%, respectively. There was high compliance in the five preceding MDA rounds in Ahanta West and Kassena Nankana West districts, both considered hotspots of lymphatic filariasis transmission.

**Conclusions:**

The study on persistent transmission of lymphatic filariasis in the two areas in Ghana present information that shows the importance of local understanding of factors affecting control and elimination of lymphatic filariasis. Unlike Kassena Nankana West district where transmission dynamics could be explained by initial infection prevalence and low vector densities, ongoing lymphatic filariasis transmission in Ahanta West district might be explained by high biting rates of *An. gambiae* and initial infection prevalence, coupled with high densities of *An. melas* and *Mansonia* vector species that have low or no teeth and exhibiting limitation.

**Electronic supplementary material:**

The online version of this article (10.1186/s40249-019-0520-1) contains supplementary material, which is available to authorized users.

## Multilingual abstract

Please see Additional file 1 for translations of the abstract into five official working languages of the United Nations.

## Background

Lymphatic filariasis is a debilitating disease affecting the health, productivity and wellbeing of infected individuals and communities [1, 2]. Over 90% of infections worldwide is caused by *Wuchereria bancrofti* and the remaining by *Brugia* species [3]. Mosquitoes belonging to the genera *Aedes*, *Anopheles*, *Coquillitedia, Culex* and *Mansonia* (depending on their geographical location) are involved in transmission [4]. In Ghana, the main vectors are *An*. *gambiae* and *An. funestus* senso lato (s.l.) and the minor are *An*. *pharoensis* [5] and *Mansonia* species [6].

It is assumed that in areas where the primary vectors are *Anopheles* species, about 5–6 rounds of mass drug administration (MDA) should be effective in breaking transmission of lymphatic filariasis [7]. This assumption did not consider confounding factors such as spatial heterogeneities which, when included in an intervention model, may give predictions that could exceed the 5–6 rounds of MDA even with > 65% MDA coverage for achieving lymphatic filariasis elimination in various endemic areas [8]. A scenario modelled by Michael et al. [8] suggested that with the current MDA regimen, Ghana is likely to eliminate lymphatic filariasis by 2020. However, the authors indicated that lymphatic filariasis transmission is focal due to a number of factors including spatial heterogeneities [8]. This therefore implies that interventions should at best consider these unique factors in each endemic foci. In Ghana, MDA commenced in five districts in the year 2000, and was scaled up to cover all endemic districts by 2006 [9]. Hence, by 2014, each endemic district had received at least eight rounds of MDA, which was expected to have interrupted transmission. However, evaluations revealed that infections still persisted in 22 districts (termed “hotspot” districts) with microfilariae (mf) prevalence greater than 1% [10].

The persistent transmission of lymphatic filariasis may be influenced by different factors [11–14]. These include pre-control lymphatic filariasis prevalence and infection intensity, population treatment coverage and compliance, vector competence and vectorial capacity and socio-cultural factors. *Wuchereria bancrofti* transmission in a vector population depends on the ability of mosquitoes to ingest and support the development of mf [15]. Importantly, mf ingested is affected by cibarial teeth, a physical barrier in the foregut of mosquitoes. This may influence the dynamics of filarial transmission and impact on control measures [16]. Additionally, the initiation of infections for *W. bancrofti* depends on the availability of vector species and high vector biting rates [17]. The success of MDA also depends on the extent of the population treatment coverage. The recommended population treatment coverage by WHO should exceed 65% of the endemic population [18]. Indeed, such MDA treatment coverage rates, coupled with effective compliance (i.e. willingness of individuals to ingest the drug), are necessary for a successful MDA programme.

In Ghana, lymphatic filariasis transmission persists in several districts, even after more than ten rounds of MDA, despite reported average treatment coverage rates of > 65%. Consequently, these districts are labelled as “hotspots”. Other districts have successfully passed a transmission assessment survey (TAS), and hence, MDA has been stopped [9]. For the current study, the latter districts are termed “control”. Our objective was to determine factors that influence the transmission of lymphatic filariasis, in selected hotspots and control districts in the Western and Upper East regions of Ghana.

## Methods

### Study sites

**Fig. 1:**
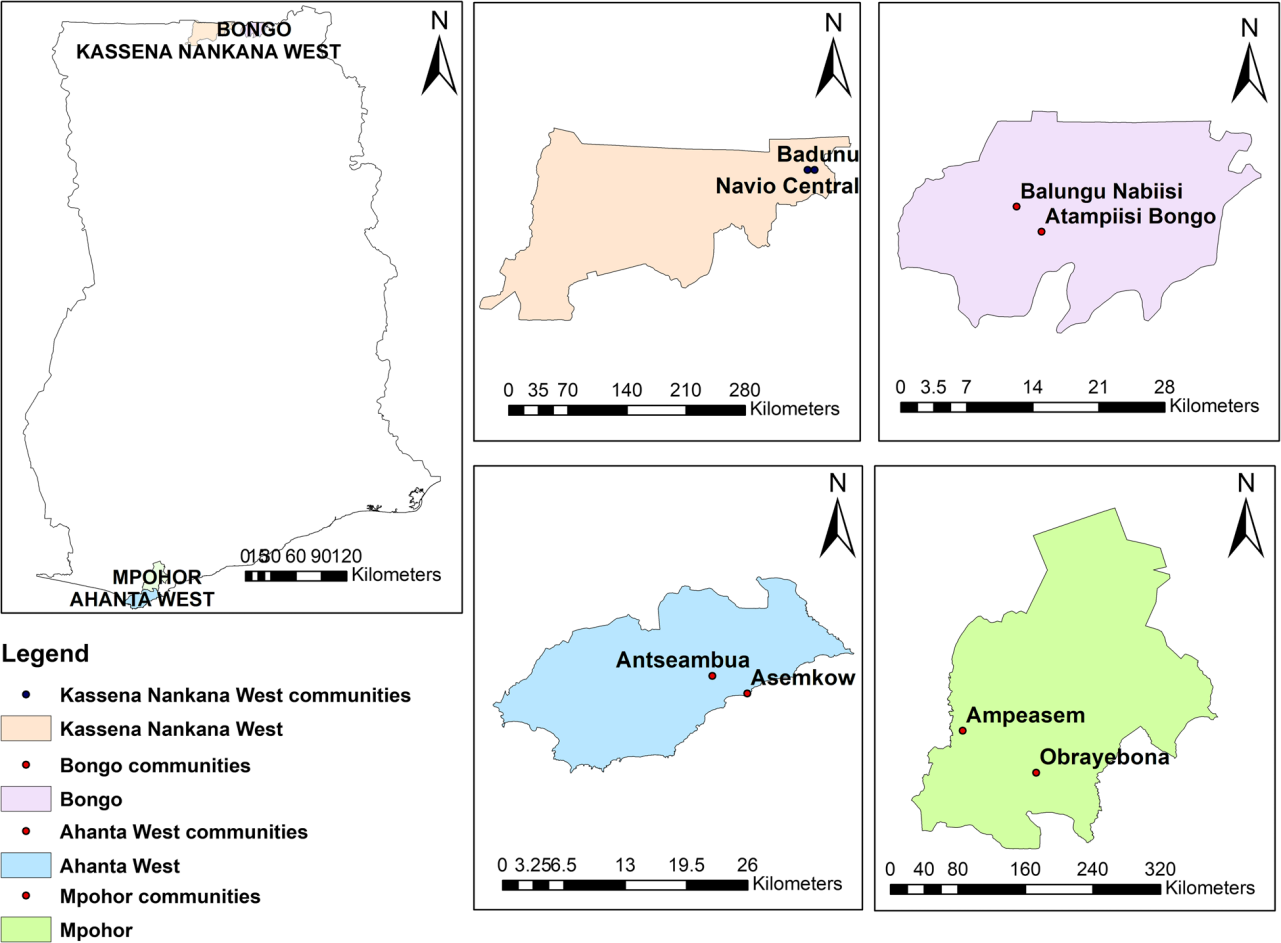
Map showing lymphatic filariasis study districts from the Western and Upper East regions of Ghana

The study was conducted in eight communities from four districts in Ghana. There were four communities in two hotspot districts; namely, Asemkow (geographical coordinates 4°82’ N latitude, 1°88’ W longitude) and Antseambua (4°85’ N latitude, 1°93’ W longitude) in the Ahanta West district; and Badunu (10°96’ N latitude, 1°06’ W longitude) and Navio Central (10°96’ N latitude, 1°05’ W longitude) in the Kassena Nankana West district. Additionally, there were four communities in two control districts; namely, Balungo Nabiisi (10°93’ N latitude, 0°84’ W longitude) and Atampiisi Bongo (10°91’ N latitude, 0°82’ W longitude) in the Bongo district and Ampeasem (5°04’ N latitude, 1°94’ W longitude) and Obrayebona (5°00’ N latitude, 1°87’ W longitude) in the Mpohor district. The Ahanta West and Mpohor districts belong to the high rain forest vegetation climatic zone, whilst Kassena Nankana West and Bongo districts have sub-Sahelian climate (Fig. 1).

### Mosquito collection and processing

Entomological surveys were conducted monthly in all the study communities. Mosquitoes were collected over a 13-month period from the beginning of July 2015 to the end of July 2016. Samples were collected using window exit traps, pyrethroid spray catches and human landing catches [17]. In each district, there were 16 community vector collectors (CVCs). Each district had two communities selected and the eight CVCs divided into two teams (four per team). Human landing catches involved two CVCs sampling indoor, and the other two outdoor in two different households simultaneously for every sampling night. Mosquitoes were collected hourly from 21:00 to 5:00 the next morning. Starting human landing catches earlier instead of the 21:00 would not have had any significant impact on the results as relatively few *An. gambiae* s.l. bite before 21:00 in the Upper East Region [19]. This time was therefore replicated in other districts to have a uniform setting. Pyrethrum spray collection was done by the CVCs from 5:00 to 8:00 in up to ten different households. Before every sampling night, two window exit traps were fixed in two different households at 18:00 and removed after 8:00 the next morning. Sampling was done twice a month in two different households every catch night in each community. All mosquitoes were identified at species level, using morphologic identification keys [20, 21].

Molecular identification was done by extracting DNA from mosquito legs using a standard protocol described by Xu et al. [22]. Sibling species of *An. gambiae* complex were identified using polymerase chain reaction (PCR), as described by Scott et al. [23]. This was followed by restriction fragment length polymorphism (RFLP) to distinguish the species *An. coluzzii* and *An. gambiae* senso stricto (s.s.) [24].

### Assessment of infection and infectivity rates in *An. gambiae*

In general, the rationale for selecting mosquitoes was aimed at having proportional numbers of mosquitoes in the various districts dissected for the estimation of infection and infectivity. Samples collected with human landing catches were used to estimate infection, infectivity and annual biting rate (ABR). For estimation of infection and infectivity rates, *An. gambiae* samples were dissected and observed for the various stages of the parasites [17].

### Cibarial armature characterisation

The heads of 224 mosquitoes (anophelines and culicines) consisting of 14 mosquitoes per species for each district were selected with reference to similar studies [11, 25, 26]. The mosquito heads were detached and placed in a 1.5 ml microcentrifuge tube containing clearing medium (consisting of equal volumes of chloral hydrate and phenol) [11]. Tubes were kept in the dark for about a week to clear the mosquito heads [11]. Clearing took longer for dark (highly melanised) mosquitoes, such as *Aedes* species (approximately one month). After clearing, the mosquito heads were placed on a clean glass slide and a drop of Puri’s (mounting) medium was added before covering with a cover slip. The heads were mounted dorso-ventrally to enhance viewing and counting of the cibarial teeth. The cibarial armature was observed under a compound microscope at X 1000 magnification. The mounted mosquito head was kept at room temperature for at least 1 week and the total number of cibarial teeth counted and recorded.

### Questionnaire survey

Our study pursued a cross-sectional design with questionnaires randomly administered to individuals in the various districts. The questionnaire sought to obtain information about treatment compliance and involvement in vector control activities in the study districts. Before this study, the questionnaire was validated using the results of a pre-test. This was to serve as a quality control.

### Statistical analysis

Data were entered using Microsoft Excel (2013 version) and imported into STATA version 11 (Stata Corporation; College Station, TX, USA). We checked for significant differences of the cibarial teeth numbers according to mosquito species, and of mosquito abundance comparing hotspot and control sites using *F*-test. Data obtained from the National Neglected Tropical Diseases Control Programme Unit pertaining to MDA coverage in the various communities within the various districts were entered in Excel and annual frequencies of MDA coverages calculated at the unit of the district. The frequencies for MDA compliance were analysed using EpiInfo version 7 (Centers for Disease Control and Prevention; Atlanta, GA, USA). *P*-values ≤0.05 were considered statistically significant. Entomological parameters assessed included:

- Infection rate: proportion of mosquitoes found infected after dissection with any *W. bancrofti* larval stage:
  [Number of mosquitoes with (mf or L_1_ or L_2_ or L_3_)] / [Number of mosquitoes dissected] × 100
- Infectivity rate: proportion of mosquitoes found infected with one or more infective larvae: [Number of mosquitoes with L_3_] / [Number of mosquitoes dissected] × 100
- ABR: estimated number of mosquitoes biting a human per year:
  [(Number of mosquitoes caught) / (Number of catchers × number of catch night)] × 365 days [17, 27, 28].

## Results

### Mosquito species composition and abundance

**Fig. 2:**
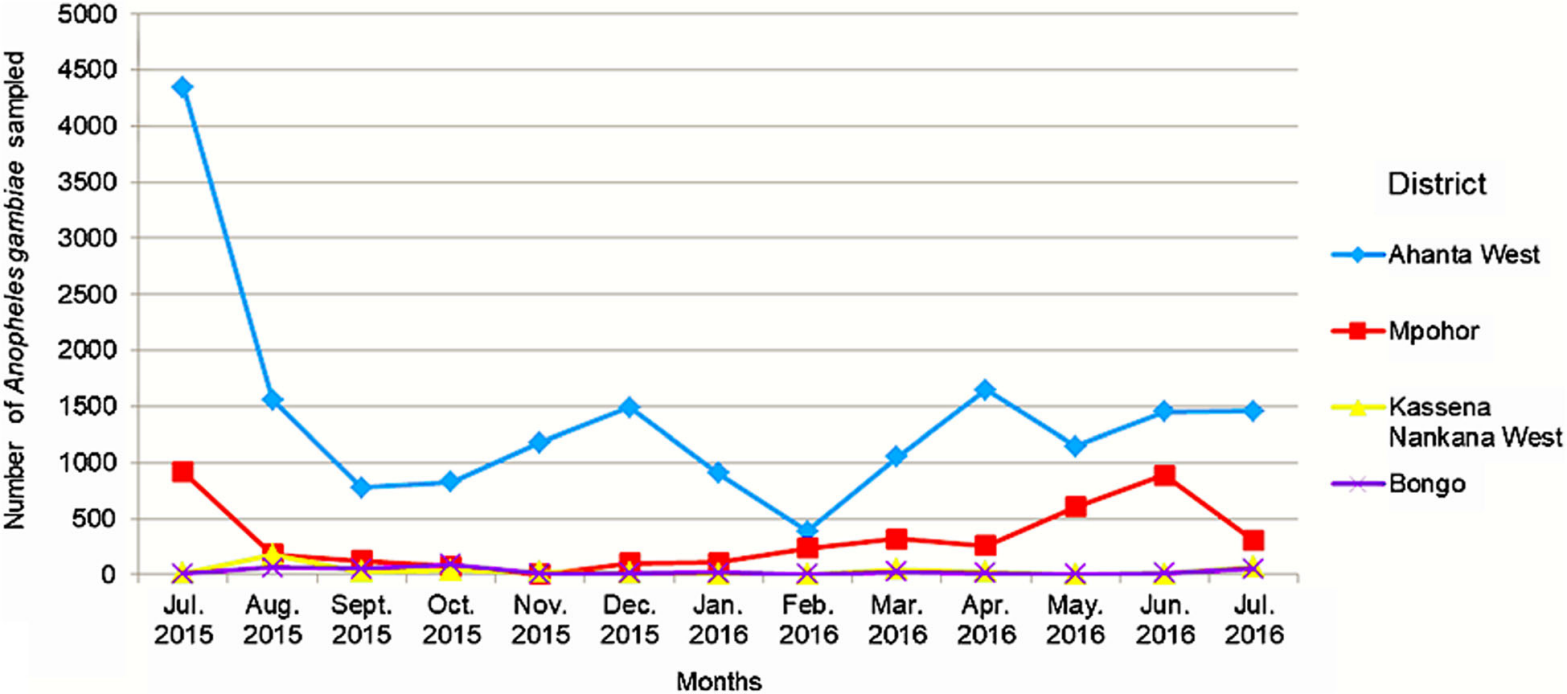
*Anopheles gambiae* sampled from Western and Upper East regions, Ghana from July 2015 to July 2016

A total of 31 064 mosquitoes were sampled from all the study areas. There was a significant difference in the number of mosquitoes collected from Ahanta West district compared to Mpohor district in the Western region (*F* = 16.09, *P* = 0.002). No difference was observed between hotspot and control districts for the Upper East (*F* = 2.16, *P* = 0.185). The mosquitoes collected in this study were *Aedes* species, *An. coustani*, *An. gambiae* s.l., *An. pharoensis*, *Culex* species and *Mansonia* species. *An. gambiae* s.l., which serves as the principal vector of lymphatic filariasis in Ghana, was the most abundant mosquito species sampled in hotspot and control districts in both the Western and Upper East regions. Relatively higher numbers were sampled from the Ahanta West district (Table 1). Figure 2 shows the total number of *An. gambiae* mosquitoes sampled for the various months from all the study areas. The ABR for *An. gambiae* mosquitoes sampled by human landing catches in Ahanta West, Mpohor, Kassena Nankana West and Bongo districts were 15 987, 3604, 376 and 306 bites per person, respectively (Table 2). There was a significant difference in ABR between Ahanta West and Mpohor districts (*F* = 15.16, *P* = 0.001), but not between Kassena Nankana West and Bongo districts (*F* = 0.13, *P* = 0.718). Mosquitoes belonging to the genus *Mansonia* were the second most abundant sampled in Ahanta West district (*n* = 2434) compared to Mpohor (*n* = 80). The Upper East region, however, had *Culex* being the second most abundant species with relatively high numbers sampled from Kassena Nankana West district (*n* = 879) compared to Bongo (*n* = 626). In Ahanta West district, more *Culex* species were collected compared to Mpohor district. Relatively low numbers of *Aedes*, *An. pharoensis* and *An. coustani* were sampled from all study areas in the Western and Upper East regions.

**Table 1:** Species composition and abundance of mosquitoes collected from the study sites

		Total number of mosquito species collected (2015–2016)							Total number of mosquitoes collected (%)	Species identified molecularly
District (hotspot/control)	Region	*Anopheles gambiae*	*Anopheles pharoensis*	*Anopheles coustani*	*Culex* Species	*Mansonia uniformis*	*Mansonia africana*	*Aedes* species		
Ahanta West (hotspot)	Western	18 880	36	4	1221	774	1660	9	22 584 (72.7)	*Anopheles coluzzii*/*Anopheles melas*
Mpohor (control)	Western	4603	10	3	81	61	19	7	4784 (15.4)	*Anopheles coluzzii*
Kassena Nankana West (hotspot)	Upper East	1239	4	13	879	9	3	44	2191 (7.1)	*Anopheles coluzzii/Anopheles arabiensis*
Bongo (control)	Upper East	826	4	2	626	3	2	42	1505 (4.9)	*Anopheles coluzzii/Anopheles arabiensis*
	Total								31 064 (100)

**Table 2:** The annual biting rates for lymphatic filariasis vectors in four districts, Ghana

Annual biting rate (ABR) (bites/person/year)				
Mosquito species	Ahanta West	Mpohor	Kassena Nankana West	Bongo
*An. gambiae*	15 987	3604	376	306
*Mansonia* species	2093	63	9	4

The annual biting rates due to human landing catches for *An. gambiae* complex and *Mansonia* species, vectors for lymphatic filariasis transmission in four districts from Ghana

Molecular identification of the *An. gambiae* complex showed that *An. gambiae* s.s., *An. melas* and *An. arabiensis* were the only species identified as sibling species. *An. arabiensis* were identified in both hotspot and control districts in the Upper East region, whilst *An. melas* were found only in Ahanta West district in the Western region. Further molecular analysis of *An. gambiae* s.s. indicated that *An. coluzzii* (previously the M form of *An. gambiae* s.s.) [29] was the only species in the study areas.

### Infection and infectivity rate for *An. gambiae* complex

A total of 1116 mosquitoes were selected for the 13 months spanning both wet and dry seasons in all districts. Ahanta West, Mpohor, Kassena Nankana West and Bongo districts had a total of 320, 368, 217 and 211 mosquitoes dissected, respectively. A total of eight mosquitoes were found positive for the various stages of the filarial parasite (L_1_, L_2_ and L_3_), with two samples being infective (L_3_). All samples found positive were *An. melas* found only in the Ahanta West district. The average infection and infectivity rates were (0.025 [2.5%], 95% *CI*: 0.8–4.2) and (0.006 [0.6%], 95% *CI*: 0.0–1.5) respectively. The presence of *W. bancrofti* was confirmed [30]. Dissected samples from Mpohor, Kassena Nankana West and Bongo districts all tested negative for filarial parasites.

### Cibarial armature characterisation

**Fig. 3:**
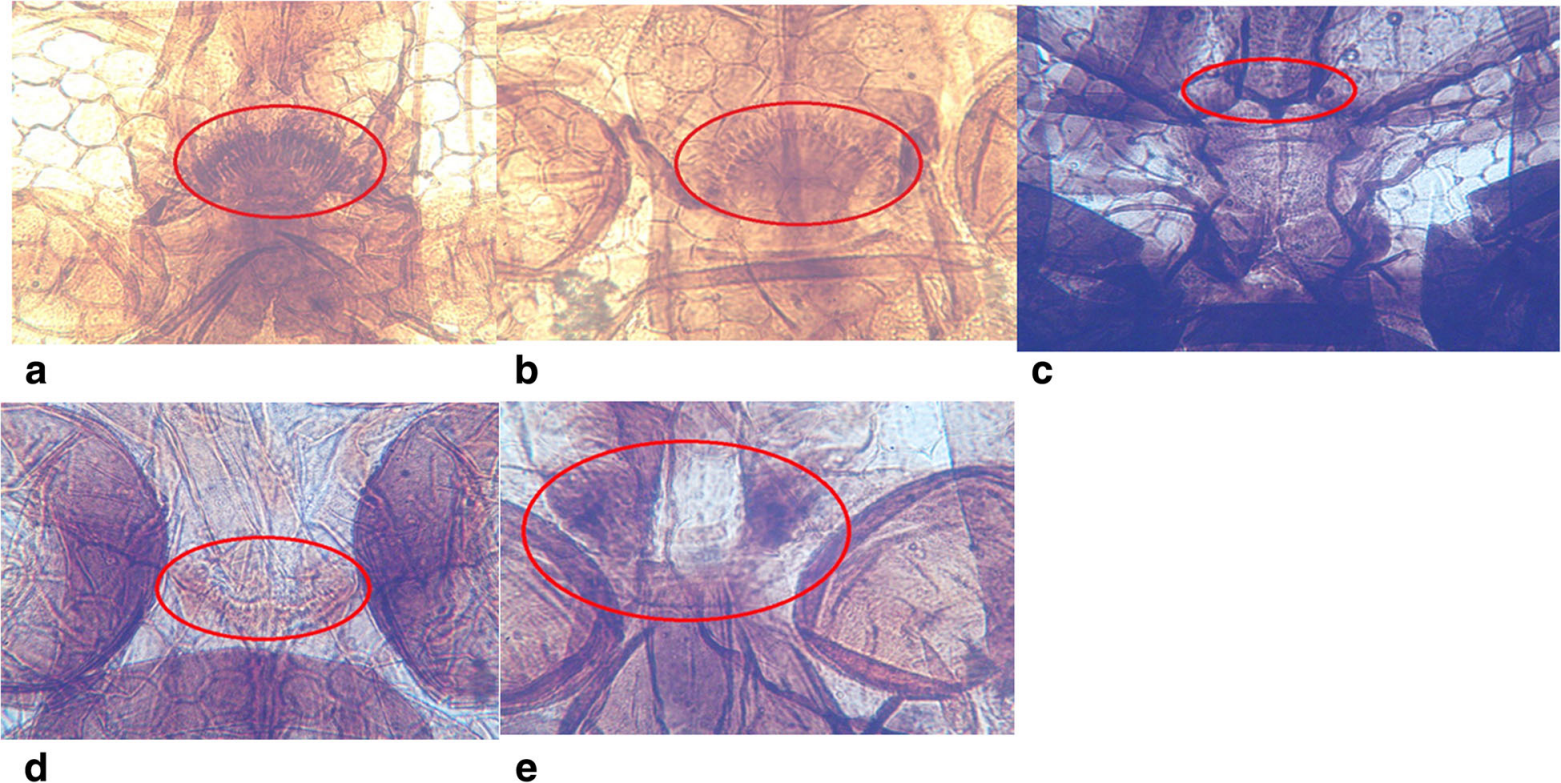
Cibarial armatures of mosquitoes from Western and Upper East regions, Ghana, July 2015 to July 2016. **a**. *An. gambiae* complex, **b**. *An. pharoensis*, **c**. *Aedes* species, **d**. *Culex* species and **e**. *Mansonia* species. The cibarial armatures of the mosquito species *Culex*, *An. gambiae* complex and *An. pharoensis* have cibarial teeth present. There are no cibarial teeth present for *Aedes* and *Mansonia* species.

Out of 224 mosquito heads processed, 140 samples properly cleared, and hence, were used for cibarial armature analysis. These samples were from both hotspot and control districts. The observation of the cibarial teeth of *An. gambiae* complex all showed that the teeth were sharp, pointed and long, but relatively fewer than that of *An. pharoensis*, which had pointed deep–rooted narrow based teeth. *Culex* species had the highest number of teeth, which were short, small sized and blunt. *Aedes*, *Ma. uniformis* and *Ma. africana* species had no cibarial teeth. The above description for the structure and shape of the cibarial teeth was similar for all mosquito species from hotspot and control districts in the two regions (Table 3). The structure of cibarial armatures of the various species are shown in Fig. 3. The mosquito species with the highest mean number of teeth was observed among *Culex* mosquitoes for both hotspot and control sites in the Western and Upper East regions, and the lowest observed in *An. melas*, which was found only in Ahanta West district (Table 3). There were no significant differences in the mean number of teeth between *An. coluzzii* (*F* = 2.12, *P* = 0.243) from hotspot and control study areas in the Western region. The same was observed for *Culex* (*F =* 3.00, *P* = 0.250) from this region. Results from Bongo and Kassena Nankana West districts also showed no significant differences in the mean number of teeth for *An. coluzzii* (*F* = 0.63, *P* = 0.277), *Culex* (*F* = 0.58, *P* = 0.231) and *An. pharoensis* (*F =* 0.57, *P* = 0.363).

**Table 3:** Mosquito heads from Western and Upper East regions, Ghana cleared and examined for cibarial armature

District (hotspot/control)	Mosquito species	Mean no. of teeth/*SD*	Median (teeth range)	Description of teeth (shape)
Ahanta West (hotspot)	*An. coluzzii*	16.0/ ± 1.0	16 (15–17)	Sharp/pointed/long
	*Culex* species	24.3/ ± 2.2	24.5 (21–27)	Small/blunt/short
	*Mansonia* species	0.0/ ± 0.0	0 (0)	Teeth absent
	*Aedes* species	0.0/ ± 0.0	0 (0)	Teeth absent
	*An. melas*	13.3/ ± 0.5	13 (13–14)	Sharp/pointed/long
Mpohor (control)	*An. coluzzii*	16.0/ ± 1.7	15 (15–18)	Sharp/pointed/long
	*Culex* species	25.2/ ± 1.4	25 (23–27)	Small/blunt/short
	*Mansonia* species	0.0/ ± 0.0	0 (0)	Teeth absent
	*Aedes* species	0.0/ ± 0.0	0 (0)	Teeth absent
Kassena Nankana West (hotspot)	*An. coluzzii*	15.8/ ± 1.8	15 (13–18)	Sharp/pointed/long
	*An. pharoensis*	21.3/ ± 1.5	21 (20–23)	Pointed/deep-rooted/narrow based
	*Culex* species	26.8/ ± 2.0	26 (25–30)	Small/blunt/short
	*Mansonia* species	0.0/ ± 0.0	0 (0)	Teeth absent
	*Aedes* species	0.0/ ± 0.0	0 (0)	Teeth absent
	*An. arabiensis*	16/ ± 0.0	16 (16)	Sharp/pointed/long
Bongo (control)	*An. coluzzii*	15.8/ ± 1.4	15 (14–18)	Sharp/pointed/long
	*An. pharoensis*	20.7/ ± 1.2	20 (20–22)	Pointed/deep-rooted/narrow based
	*Culex species*	25.8/ ± 2.7	24 (24–30)	Small/blunt/short
	*Mansonia* species	0.0/ ± 0.0	0 (0)	Teeth absent
	*Aedes* species	0.0/ ± 0.0	(0)	Teeth absent
	*An. arabiensis*	16/ ± 0.0	16 (16)	Sharp/pointed/long

### MDA coverage and baseline (pre-intervention) mf and antigenaemia prevalence

**Fig. 4:**
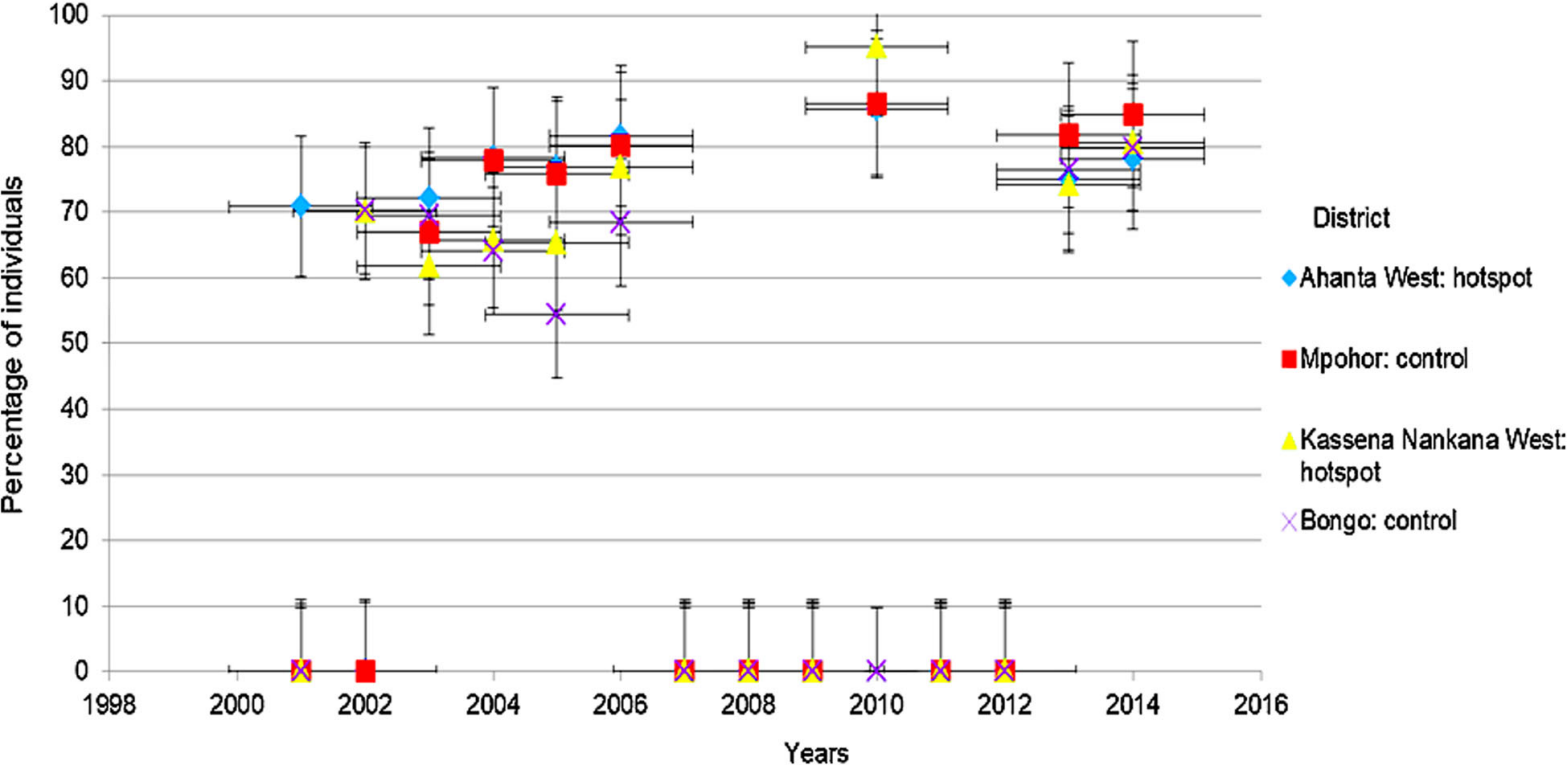
MDA coverage for hotspot and control districts in the Western and Upper East regions, Ghana

Analysis of MDA coverage data showed that the treatment coverage for the various years in both Ahanta West and Mpohor districts were above 65%. However, in the Upper East region, Kassena Nankana West and Bongo districts had greater than 65% MDA coverage for all years indicated except in 2003 for Kassena Nankana West and 2004/5 for Bongo districts (Fig. 4). By 2016, Ahanta West, Mpohor, Bongo and Kassena Nankana West districts had been involved in 16, 11, 13 and 15 rounds of MDA, respectively. However, there were no MDA data for some of the years (from 2000 to 2014) in all the districts. Data were absent for Mpohor, Kassena Nankana West and Bongo districts for 2001. Ahanta West/Mpohor and Bongo had no data for the years 2002 and 2010, respectively. All districts, however, had no data for 2007, 2008, 2009, 2011 and 2012.

A retrospective assessment of baseline mf and antigen prevalence for the various districts showed high baseline mf and antigenaemia prevalence for all districts, except Mpohor where zero prevalence was reported for both mf and antigen. The baseline mf and antigen prevalence for Ahanta West, Kassena Nankana West and Bongo districts were 19.5 and 32.8%, 29.4 and 45.3%, 16.7 and 21.2%, respectively (Table 4).

**Table 4:** Baseline microfilariae and antigenemia prevalence from the Ghana NTD Programme

District (hotspot/control)	Baseline mf prevalence (year)	Baseline antigen prevalence (year)
Ahanta West (hotspot)	19.5% (2000)	32.8% (2000)
Mpohor (control)	0 (2000)	0 (2000)
Kassena Nankana West (hotspot)	29.4% (2000)	45.3% (2000)
Bongo (control)	16.7% (2004)	21.2% (2004)

### Demographic characteristics

Questionnaires from 438 individuals (229 females, 209 males) were analysed in the four districts from the Western and Upper East regions. The age distribution of the respondents ranged from 15 to 92 years (mean = 37.4 years; median = 35 years). Half of the respondents were farmers (*n* = 220; 50.2%), 62 were fishermen (14.2%), while 26 were unemployed (5.9%) or involved in other occupations (*n* = 130; 29.7%).

### MDA compliance

**Fig. 5:**
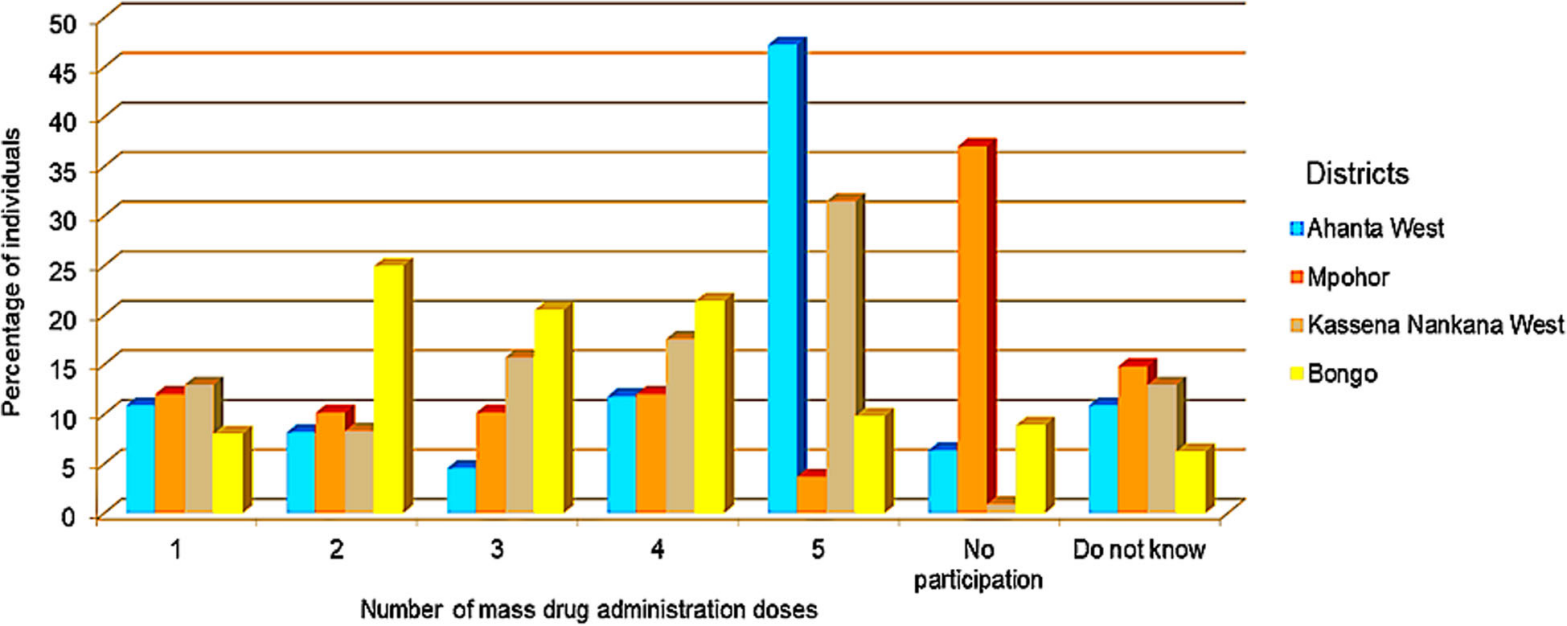
Compliance to last five MDA doses in study districts, Western and Upper East, regions, Ghana

Questionnaire data showed that out of the 110, 108, 108 and 112 respondents from Ahanta West, Mpohor, Kassena Nankana West and Bongo districts, 90.0, 53.7, 87.0 and 89.3%, respectively, affirmed their participation in MDA activities. In relation to MDA compliance, the percentages of individuals shown to have complied with the previous five rounds of MDA were 47.3, 3.7, 31.5 and 9.8% for Ahanta West, Mpohor, Kassena Nankana West and Bongo districts, respectively. Our results revealed that relatively high proportion of individuals from Mpohor district did not participate in MDA activities (Fig. 5).

### Vector control

Information on vector control activities from respondents in our four study districts indicated that bednet usage and indoor residual spraying were relatively high: 69.1–91.1% for bednet and 38.9–85.5% for indoor residual spraying.

## Discussion

It is estimated that for the interruption of lymphatic filariasis transmission, microfilariae prevalence should be less than 1% or antigen prevalence less than 2% [18]. These criteria are used for the roll out of intervention programmes in all lymphatic filariasis endemic regions. In Ghana, control of lymphatic filariasis by means of MDA has been going on for almost two decades. At the time of the current study in 2016, most endemic communities should have interrupted transmission and began transmission assessment survey (TAS) or post-MDA surveillance. However, there are endemic foci still having transmission even after several rounds of MDA [10]. Mathematical model simulations suggest that different countries may have different mf breakpoints for interruption of lymphatic filariasis [8]. Hence, there is a need to have a critical look at the 1% mf or 2% antigen thresholds used in various endemic regions for interruption of transmission. The reasons contributing to this persistent transmission are not clear. Vector species and abundance [17], vector control activities [31], vector competence, MDA compliance and therapeutic coverage [12], drug efficacy [32] and possible vector-parasite combinations [33] are important factors that govern the transmission of lymphatic filariasis. However, in any particular situation either all or some of these factors may be important and need to be understood to resolve any ongoing transmission.

Results derived from the current study showed that, with the exception of *An. melas*, mosquito species composition was similar in hotspot and control districts. However, higher numbers of mosquitoes were obtained from hotspots, compared to control districts in the same ecological zone. The transmission of lymphatic filariasis is significantly influenced by vector density [17]. The consistent high number of mosquitoes collected from Ahanta West compared to Mpohor district might be contributing to the persistence of lymphatic filariasis transmission in Ahanta West district after several rounds of MDA. Additionally, on-going lymphatic filariasis transmission in Kassena Nankana West district might be explained by the relatively high number of mosquitoes collected in this district, compared to Bongo.

Vector-parasite density dependent relationships of limitation, stable transmission of lymphatic filariasis even at low mf levels, and facilitation, transmission of lymphatic even at high mf levels [19, 34], are known to influence elimination of lymphatic filariasis. Members of the *An. gambiae* are generally considered to exhibit facilitation and hence at low mf levels are not efficient. It is expected that with *An. gambiae* serving as major vector, lymphatic filariasis should have been eliminated in these districts. *An melas*, which is part of the *An. gambiae* complex, has been shown to exhibit limitation [11, 19, 35], and hence, able to pick mf at low parasitaemia and sustain their development to the infective stage. *An. melas* observed only in Ahanta West district might explain why transmission has been sustained, though at low mf levels.

Additionally, *Mansonia* species are known to exhibit limitation [1]. Higher numbers of this species were sampled from Ahanta West than Mpohor District, and very few in Kassena Nankana West and Bongo districts. *Mansonia* species have been incriminated as one of the vectors involved in lymphatic filariasis transmission in Ghana [6]. While *Mansonia* were not examined for *W. bancrofti* in this study, its presence in relatively high numbers in Ahanta West district could also be an additional factor sustaining the transmission of lymphatic filariasis in this area. *Culex* mosquitoes had higher numbers sampled in Ahanta West compared to Mpohor district, while similar numbers were collected in Kassena Nankana West and Bongo districts. *Culex* mosquitoes exhibit limitation [1] and transmit lymphatic filariasis in East Africa [6]. Appawu et al. [27] showed that *Culex* species in Ghana are refractory to *W. bancrofti* and do not support their development to the infective stage. However, studies in Nigeria [36, 37], showed that *Culex* transmit lymphatic filariasis.

Cibarial teeth in mosquitoes act as a physical barrier and influence the transmission dynamics of lymphatic filariasis. The cibarial teeth number and shape influence mf intake by inflicting lacerations on ingested parasites [11, 15]. However, more *Mansonia* species, lacking cibarial teeth and competent vectors at low parasitaemia were collected in Ahanta West. Furthermore, *An. melas* with relatively fewer cibarial teeth numbers was found in Ahanta West, while this species was absent in Mpohor district. All mosquito species common to Ahanta West, Mpohor, Kassena Nankana West and Bongo districts had similar cibarial teeth numbers and shape.

The residual transmission of lymphatic filariasis in an area may be influenced by differences in the distribution of vectors [38]. In our study, *An. melas* was found only in Ahanta West district. Another factor is the vector-parasite combinations to lymphatic filariasis infection at low mf prevalence. *An. gambiae* complex exhibit facilitation but *An. melas* belonging to this complex exhibit limitation. This may account for differences in transmission potential within the *An. gambiae* complex [4]. The competence of *An. melas* to *W. bancrofti* infection at low mf prevalence will contribute to persistent lymphatic filariasis transmission. As suggested by our dissection data, the presence of L_3_ in *An. melas* proves its involvement in ongoing transmission of lymphatic filariasis in Ahanta West district.

Analyses of MDA coverage data obtained from the National Neglected Tropical Disease Control Programme revealed at least 65% MDA coverage for all the districts. It has been hypothesised that annual MDA with adequate consistent coverage of at least 65% should make elimination possible [18]. This hypothesis was based on early models for implementing MDA intervention programmes without possibly considering spatial heterogeneities. Spatial heterogeneities when adopted by intervention models may give predictions that could exceed the 5–6 rounds of MDA recommended to interrupt lymphatic filariasis transmission. This in turn lengthens the period needed for achieving lymphatic filariasis elimination at a given endemic area. For Ghana, it was predicted that lymphatic filariasis could be interrupted by 2020 as revealed by mathematical modelling [8]. The authors however suggested that lymphatic filariasis transmission is focal due to a wide range of factors in endemic areas [8]. This therefore implies that intervention programmes rolled out in endemic areas should be specific and targeted in each endemic foci.

Community compliance to MDA is important in understanding persistent transmission of lymphatic filariasis. The evaluation of the districts’ participation in the previous five rounds of MDA indicated a higher percentage of respondents from Ahanta West district (47.3%) and Kassena Nankana West district (31.5%), reporting to have taken the drugs all five times, compared to much lower rates in Mpohor (3.7%) and Bongo (9.8%). Thus the ongoing transmission of lymphatic filariasis in Ahanta West may not be due to MDA compliance, but driven by other factors.

The results from this study indicated higher bednet usage among community members in control compared to hotspot districts. This observation may have contributed to the control of lymphatic filariasis in the control districts. In Gambia, for example, Rebollo and colleagues observed that interruption of lymphatic filariasis transmission could have possibly been due to the extensive national bednet usage for malaria control [31, 39]. It should be noted that Gambia used bednets without MDA and at present, there is no explanation on how lymphatic filariasis was eliminated aside bednet usage. Indoor residual spraying activities in all districts were high, except for Mpohor district. AngloGold Ashanti Malaria Control Ltd., a subsidiary of AngloGold Ashanti (AGA), from 2013 to 2015 conducted indoor residual spraying activities twice yearly in about 40 districts in Ghana. Due to limited resources, indoor residual spraying was done only in districts with high malaria prevalence, excluding Mpohor (unpublished data, AGA). However, it is possible that other private agencies aside AGA sprayed a few communities in Mpohor, explaining the low percentage of respondents (38.9%) affirming indoor residual spraying activities. While the indoor residual spraying data in the Western Region may not be sufficient to draw conclusions, the results from the Upper East Region on the other hand, indicate that the lower vector control activities in Kassena Nankana West compared to Bongo District could be a possible indicator for control of lymphatic filariasis transmission in control districts. Thus, supporting the important role vector control plays in the control of lymphatic filariasis [40].

There were a couple of limitations to this study. First, Mpohor was selected as a control district, although retrospective analysis of data revealed a zero prevalence at the inception of MDA in the year 2000. A study site with prevalence similar to Ahanta West district and with successful MDA treatment history would have been preferable. Second, the MDA data collected by the National Neglected Tropical Disease Control Programme could not be verified. An earlier study has shown MDA data reported by the programme to be inaccurate [41]. There were also some missing MDA data for some of the years in all the study districts.

## Conclusions

The Global Programme to Eliminate Lymphatic Filariasis (GPELF) aims at interrupting lymphatic filariasis transmission. This is based on an estimated duration of 5 years at 1% mf prevalence, which might not be feasible in all endemic areas. It is important to understand the local factors responsible for persistent transmission of lymphatic filariasis in a given area. In our study areas, transmission of lymphatic filariasis in hotspots despite many years of treatment could not be attributed to low MDA coverage and compliance when compared to control districts. In Ahanta West district, our data suggests high biting rates of vector species in the *An. gambiae* complex, initial infection prevalence rates and low vector control to ongoing lymphatic filariasis transmission. Additionally, the presence of *An. melas* and *Mansonia*, with less or no cibarial teeth may further contribute to transmission. In Kassena Nankana West district, transmission dynamics could be explained by the presence of relatively low numbers and biting rates of *An. gambiae* complex together with initial infection prevalence as reported by our study. Furthermore, low densities of *Mansonia* and the absence of *An. melas* may be reasons why no infections were recorded in this district.

## Additional file

Additional file 1: Multilingual abstracts in the five official working languages of the United Nations. (PDF 262 kb)

## Abbreviations

ABR: Annual biting rate
AGA: AngloGold Ashanti
CVC: Community vector collector
MDA: Mass drug administration
mf: Microfilariae
PCR: Polymerase chain reaction
RFLP: Restriction fragment length polymorphism
s. l: Senso lato
s. s: Senso stricto
TAS: Transmission assessment survey
