# Supplementary material for: Potential factors influencing lymphatic filariasis transmission in “hotspot” and “control” areas in Ghana: the importance of vectors
Source: Infect Dis Poverty. 2019 Feb 5;8:9. doi: 10.1186/s40249-019-0520-1 (PMC6362603; doi:10.1186/s40249-019-0520-1)
Supplement: Supplementary file 1 — Multilingual abstracts in the five official working languages of the United Nations. (PDF 262 kb) [file 40249_2019_520_MOESM1_ESM.pdf]

Translation of the abstract into the five official working languages of the United Nations

تقييم للعوامل المحتملة المؤثرة على انتقال داء الفيلاريات اللمفاوية في مناطق "النقاط المهمة" و "السيطرة" في غانا: أهمية نواقل المرض

سيلاسي بي-بانسا، جوزيف هارولد نياركو اوساي، كوادوو كيريميه فريمبونغ، اليزابيث الحسن، اوساي كويكو اكووكو، ديفيد اغيمانغ، كولينز اهورلو، ماكسويل الكسندر ابايو، بنجامين غيبيهي كويدو، مايكل دافيد ويلسون،[3]دجيدزروم كومي دي سوزا، صامويل كويكو دادزي، يورغ تزنغر، دانيال ادغي بواكوي

ملخص

معلومات أساسية: ما زالت برامج الإدارة الجماعية للأدوية (MDA) لمكافحة داء الفيلاريات اللمفاوية في غانا، مستمرة في بعض المناطق الموبوءة منذ 16 سنة. تهدف الدراسة إلى تقييم العوامل التي يمكن أن تؤثر على نجاح برامج الإدارة الجماعية للأدوية (MDA) لوقف انتقال داء الفيلاريات اللمفاوية في غانا.

الطرق: أُجريت الدراسة في منطقتين من المناطق المهمة (غرب أهانتا وغرب كاسينا نانكانا) ومقاطعتين للسيطرة على المرض (مبهور وبونغو) في غانا. ولقد تم جمع البعوض وتشخيصه باستخدام الأدوات المورفولوجية والجزيئية. تم فحص نسبة من الأغلفة الواقية للتجويف الفمي لكل نوع. تم إجراء التشريح على أنوفيلة غامبية للكشف عن الدودة الفيلارية. كان يتم إجراء استبيان للحصول على معلومات عن امتثال الإدارة الجماعية للأدوية وأنشطة مكافحة نواقل المرض. تمت مقارنة البيانات بين المناطق لتحديد العوامل التي قد تفسر الانتقال المستمر لداء الفيلاريات اللمفاوية.

النتائج: تم أخذ عينات كبيرة من البعوض من منطقة غرب أهانتا مقارنة بمنطقة مبهور (F = 16.088, P = 0.002). لم يكن هناك فرق كبير بين أعداد البعوض الذي تم جمعه في مقاطعتي غرب كاسينا نانكانا وبونغو (F = 2.159 P = 0.185). حشرة المَنْسونِيَّة كانت النوع الأكثر انتشارًا في منطقة غرب أهانتا. بعوضة An. coluzzii كان هذا النوع من البعوض منتشرًا في جميع المناطق. بعوضة An. melas وتحمل ديدانًا فيلارية مُصَابة ومُعدية وتوجد فقط في مقاطعة غرب أهانتا. لم يتم العثور علي أي اختلافات في أعداد أسنان التجويف الفمي وشكل أنواع البعوض في المناطق التي شملتها الدراسة. كانت تغطية العلاج المُبلغ عنها عالية في كافة المناطق. كان متوسط استخدام الناموسيات والرش الموضعي بالأماكن المغلقة 82.4% و66.2% على التوالي. كان هناك امتثال كبير في علاجات الإدارة الجماعية للأدوية (MDA) الخمسة السابقة في مقاطعتي غرب أهانتا وغرب كاسينا نانكانا، وكلاهما يعتبران بؤرتان لانتقال داء الفيلاريات اللمفاوية.

استنتاجات: تقدم الدراسة حول الانتقال المستمر لداء الفيلاريات اللمفاوية في المنطقتين في غانا معلومات توضح أهمية الفهم المحلي للعوامل المؤثرة في القضاء على داء الفيلاريات اللمفاوية. وعلي عكس منطقه غرب كاسينا نانكانا، حيث يمكن تفسير ديناميات الانتقال بالانتشار الأولي للعدوى وبكثافات النواقل المنخفضة، فإنه يمكن تفسير انتقال داء الفيلاريات اللمفاوية المستمر في منطقه غرب أهانتا بسبب ارتفاع معدلات اللَدْغ من بعوضة الأنوفلية الغامبية وانتشار العدوى الأولية، إلى جانب الكثافة العالية لبعوضة An. melas والأنواع الناقلة من بعوضة المَنْسونِيَّة ذات الأسنان المنخفضة أو الخالية من الأسنان وعرض القصور.

Translated from English version into Arabic by Veromarie (Veronique) and Suzanne Assenat, through

TRANSLATORS WITHOUT BORDERS

加纳淋巴丝虫病热点地区和控制区的潜在影响传播因素：评估媒介的重要性

Sellase Pi-Bansa, Joseph Harold Nyarko Osei, Kwadwo Kyeremeh Frempong, Elizabeth Elhassan, Osei Kweku Akuoko, David Agyemang, Collins Ahorlu, Maxwell Alexander Appawu, Benjamin Guibehi Koudou, Michael David Wilson, Dziedzom Komi de Souza, Samuel Kweku Dadzie, Jürg Utzinger and Daniel Adjei Boakye

.

摘要

引言：在加纳一些淋巴丝虫病流行地区，已经开展了 16 年的大规模群体服药(MDA)项目。本研究旨在评估可能影响 MDA 方案在加纳成功阻断淋巴丝虫病传播的因素。

方法：这项研究分别在加纳的两个热点地区(Ahanta West 和 Kassena Nankana West)和两个控制区(Mpohor 和 Bongo)进行。本研究利用形态学和分子工具采集和鉴定蚊媒。对每一种蚊媒的进行了一定比例的食窦检测。解剖冈比亚按蚊检测是否携带丝虫。开展问卷调查以获取 MDA 依从性和媒介控制活动的信息。最后比较各区间的数据，以确定淋巴丝虫病持续传播的因素。

方法：与 Mpohor 区相比，Ahanta 西区的蚊媒数量较多(F = 16.088, P = 0.002)。在 Kassena Nankana West 和 Bongo 地区收集的蚊媒数量没有显著差异(F = 2.159, P = 0.185)。曼蚊属主要分布在 Ahanta West。*Coluzzii* 按蚊普遍分布于四个区域。感染丝虫病的 *melas* 按蚊和感染性丝虫仅见于 Ahanta West。在研究区域，食窦牙的数量和形状没有明显差异。在所有地区，报告的治疗覆盖率都很高。平均使用蚊帐率和室内残留喷洒率分别为 82.4%和 66.2%。Ahanta West 和 Kassena Nankana West 地区均被认为是淋巴丝虫病传播的热点地区，虽然在之前的 5 次 MDA 治疗中依从性均较高。

结论：本文研究了在加纳两个淋巴丝虫病地区造成其持续传播的影响因素。研究表明，理解影响消除淋巴丝虫病的传播因素至关重要。初始感染流行率和低媒介密度是影响 Kassena Nankana West 淋巴丝虫病传播动力学的主要因素。Ahanta West 的淋巴丝虫病传播的主要影响因素是冈比亚安稳高叮人率、初始感染流行率，其他影响因素包括 *melas* 按蚊的高密度，曼蚊属没有食窦牙或者数量较少及其局限性分布。

Translated from English version into Chinese by Xin-Yu Feng, edited by Jin Chen

CHINESE CENTER FOR DISEASE CONTROL AND PREVENTION
NATIONAL INSTITUTE OF PARASITIC DISEASES

Évaluation des facteurs susceptible d’influencer la transmission de la filariose lymphatique dans des zones « sensibles » et « de référence » au Ghana : importance des vecteurs.

Sellase Pi-Bansa, Joseph Harold Nyarko Osei, Kwadwo Kyeremeh Frempong, Elizabeth Elhassan, Osei Kweku Akuoko, David Agyemang, Collins Ahorlu, Maxwell Alexander Appawu, Benjamin Guibehi Koudou, Michael David Wilson[3], Dziedzom Komi de Souza, Samuel Kweku Dadzie, Jürg Utzinger et Daniel Adjei Boakye

Résumé

Contexte : Des programmes de distribution de masse de médicaments (MDA) contre la filariose lymphatique sont en place depuis 16 ans au Ghana, dans certains districts d’endémie. Notre étude visait à évaluer les facteurs susceptibles d’avoir une incidence sur la réussite des programmes de MDA dans la lutte contre la transmission de la filariose lymphatique au Ghana.

Méthodes : L’étude a été réalisée dans deux zones sensibles (districts d’Ahanta Ouest et de Kassena Nankana Ouest), ainsi que dans deux districts de référence (Moohor et Bongo) au Ghana. Des moustiques ont été collectés et identifiés à l’aide de méthodes morphologiques et moléculaires. Une partie des armatures cibariales de chaque espèce a été examinée. Des spécimens d’*An. gambiae* ont été disséqués à la recherche de filaires. Un questionnaire a été distribué afin d’obtenir des informations concernant l’application des programmes de distribution de masse de médicaments et des activités de lutte contre les vecteurs de la maladie. Les données ont été comparées entre les districts afin de déterminer les facteurs qui pourraient expliquer la persistance de la transmission de la filariose lymphatique.

Résultats: Un nombre très élevé de moustiques dans le district d’Ahanta Ouest par rapport au district de Mpohor (F = 16,088, P = 0,002). Aucune différence significative n’a été observée entre les nombres de moustiques collectés dans le districts de Kassena Nankena Ouest et de Bongo (F = 2,159, P = 0,185). Une prévalence des espèces de *Mansonia* a été observée dans le district d’Ahanta Ouest. Les moustiques *An. coluzzii* prédominaient dans tous les districts. Des *An. melas* porteurs de filaires ingérées et infestantes ont été détectés seulement dans le district d’Ahanta Ouest. L’examen des dents cibariales des espèces de moustiques présentes dans les districts étudiés n’a révélé aucune différence de nombre ni de forme. La couverture thérapeutique était élevée dans tous les districts. Le taux d’utilisation moyenne des moustiquaires était de 82,4 % et celui des pulvérisations intérieures d’insecticide à effet rémanent de 66,2 %. Les cinq précédents programmes de distribution de masse de médicaments ont reçu une très forte adhésion des populations dans le district d’Ahanta Ouest et celui de Kassena Nankana, considérés tous deux comme des zones sensibles de transmission de la filariose lymphatique.

Conclusions : Les résultats de l’étude sur la persistance de la transmission de la filariose lymphatique dans les deux districts au Ghana soulignent qu’il importe de bien comprendre les facteurs locaux susceptibles d’influer sur l’élimination de la filariose lymphatique. Contrairement au district de Kassena Nankana Ouest, où les dynamiques de transmission peuvent s’expliquer par la prévalence initiale de l’infection et par les petites populations d’espèces vectrices, la transmission actuelle de la filariose lymphatique dans le district d’Ahanta Ouest pourrait résulter de taux élevés de piqûres de l’espèce *An. gambiae* et de la prévalence initiale de l’infection, associés à des populations importantes des espèces vectrices *An. melas* et *Mansonia*, qui ont peu ou pas de dents cibariales et présentent une limitation du passage des microfilaires.

Translated from English version into French by Nader Hassan and Ali Mohamed Magdy, through

TRANSLATORS WITHOUT BORDERS

Оценка потенциальных факторов, влияющих на распространение лимфатического филяриатоза в «горячих точках» и «контрольных» районах Ганы: важность борьбы с переносчиками инфекции

Силасси Пи-Банса (Sellase Pi-Bansa), Джозеф Харольд Ньяко Осей (Joseph Harold Nyarko Osei), Квадво Киереми Фремпон (Kwadwo Kyeremeh Frempong), Элизабет Элхассан

(Elizabeth Elhassan), Осей Квеки Акуоко (Osei Kweku Akuoko), Дэвид Аджимэн (David Agyemang), Коллинс Ахорлу (Collins Ahorlu), Максвелл Александр Арраву (Maxwell Alexander Appawu), Бенджамин Гуйбехи Коудоу (Benjamin Guibehi Koudou), Майкл Дэвид Вилсон (Michael David Wilson)[3], Дзиедром Коми де Суза (Dziedzom Komi de Souza), Сэмюел Квеку Дадзи (Samuel Kweku Dadzie), Юрг Утцингер (Jürg Utzinger) и Даниел Аджей Боаки (Daniel Adjei Boakye)

Аннотация

Вводная информация: Программы массового медикаментозного лечения (ММЛ) для борьбы с лимфатическим филяриозом в Гане проводились в некоторых эндемичных районах в течение 16 лет. Исследование было направлено на оценку факторов, которые могут повлиять на успех программ ММЛ по борьбе с распространением лимфатического филяриатоза в Гане.

Методы: Исследование было проведено в двух горячих точках (Аханта Уэст и Кассена-Нанкана Уэст) и в двух контрольных районах (Мпохор и Бонго) в Гане. Москитов собирали и идентифицировали с использованием морфологических и молекулярных инструментов. Была исследована доля москитов каждого вида с зубами в области ротоглотки. Были выполнены диссекции *An. gambiae* для обнаружения филарии. Был проведен опрос для получения информации о выполнении ММЛ и мероприятий по борьбе с переносчиками инфекции. Данные сравнивались между районами для определения факторов, которые могут объяснить постоянное распространение лимфатического филяриатоза.

Результаты: Большое количество москитов было отобрано в районе Аханта Уэст по сравнению с районом Мпохор (F = 16,088; P = 0,002). Не было значительной разницы между количеством москитов, собранных в районах Кассена-Нанкана Уэст и Бонго (F = 2,159; P = 0,185). Вид *Mansonia* преобладал в районе Аханта Уэст. Москиты вида *An. coluzzii* преобладали во всех районах. Москиты вида *An. melas* с инфицированными и инфекционными филариями были обнаружены только в районе Аханта Уэст. Не было обнаружено различий в количестве и форме ротоглоточных зубов между видами комаров в обследованных районах. Отмеченный охват лечением был высоким во всех районах. Среднее использование противомоскитных кроватных сеток и распыление инсектицидов остаточного действия внутри помещений составило 82,4% и 66,2% соответственно. Показатели выполнения пяти предыдущих видов лечениях ММЛ были высокими в районах Аханта Уэст и Кассена-Нанкана Уэст, при этом оба они считались горячими точками распространения лимфатического филяриатоза.

Выводы: В результате исследования постоянного распространения лимфатического филяриатоза в двух районах Ганы была получена информация, которая демонстрирует важность местного понимания факторов, влияющих на устранение лимфатического филяриатоза. В отличие от района Кассена-Нанкана Уэст, где динамику распространения можно объяснить начальной распространенностью инфекции и низкой плотностью популяции переносчиков инфекции, продолжающееся распространение лимфатического филяриатоза в районе Аханта Уэст может быть объяснено высокими показателями укусов видом *An. gambiae* и начальной распространенностью инфекции, наряду с большой плотностью популяции переносчиков инфекции *An. melas* и *Mansonia*, у которых нет или почти нет зубов, и которые демонстрируют другие ограничения.

Translated from English version into Russian by Oksana Weiss and Natalia Potashnik, through

Evaluación de los posibles factores influyentes en la transmisión de la filariasis linfática en zonas "activas" y "de control" en Ghana: la importancia de los vectores.

Sellase Pi-Bansa, Joseph Harold Nyarko Osei, Kwadwo Kyeremeh Frempong, Elizabeth Elhassan, Osei Kweku Akuoko, David Agyemang, Collins Ahorlu, Maxwell Alexander Appawu, Benjamin Guibehi Koudou, Michael David Wilson[3], Dziedzom Komi de Souza, Samuel Kweku Dadzie, Jürg Utzinger and Daniel Adjei Boakye

Resumen

Antecedentes: Los programas de administración masiva de fármacos (MDA por sus siglas en inglés) para el control de la filariasis linfática en Ghana llevan 16 años en funcionamiento en algunos distritos endémicos. El presente estudio se enfoca en la evaluación de los factores que podrían afectar al éxito de los programas MDA para interrumpir la transmisión de la filariasis linfática en Ghana.

Métodos: El estudio se llevó a cabo en dos zonas activas (Ahanta Oeste y Kasena Nankana Oeste) y dos distritos de control (Mpohor y Bongo) en Ghana. La recolección e identificación de los mosquitos se llevó a cabo mediante el uso de herramientas morfológicas y moleculares. Se examinó una parte de las armaduras cibariales de cada especie. Se realizaron disecciones en mosquitos *An. gambiae* para la detección de filarias. Se proporcionó un cuestionario para obtener información sobre el cumplimiento de los programas MDA y las actividades de control de vectores. Se compararon los datos obtenidos en los diferentes distritos para determinar los factores que pudieran explicar la transmisión persistente de la filariasis linfática.

Resultados: El número muestras de mosquitos obtenido en Ahanta Oeste fue superior en comparación a las recogidas en el distrito de Mpohor (F=16 088, P = 0,002). No hubo una diferencia significativa entre el número de mosquitos recogidos en los distritos de Kassena Nankana Oeste y Bongo (F = 2 159, P = 0,185). La especie *Mansonia* fue la predominante en el distrito de Ahana Oeste. Los mosquitos *An. coluzzii* fueron los más abundantes en todos los distritos. Los mosquitos *An. melas* con filarias infectadas e infecciosas solo se hallaron en el distrito de Ahanta Oeste. No se encontraron diferencias en el número y la forma de los dientes cibariales por especie de mosquito en los distritos sometidos a estudio. La cobertura de tratamiento registrada fue elevada en todos los distritos. El uso promedio de mosquiteras y rociado residual intradomiciliario fue de un 82,4 % y un 62,2 % respectivamente. El grado de cumplimiento de los cinco tratamientos MDA precedentes en los distritos de Ahanta Oeste y Kasena Nankana Oeste, ambas consideradas áreas activas de transmisión de filariasis linfática, fue elevado.

Conclusiones: El estudio sobre la transmisión persistente de la filariasis linfática en las dos áreas de Ghana presenta información que muestra la importancia del entendimiento local de los factores

influyentes en la eliminación de la filariasis linfática. A diferencia del distrito de Kasena Nankana Oeste, donde la dinámica de las transmisiones podría explicarse con la prevalencia inicial de la infección y la baja densidad vectorial, la transmisión constante de filariasis linfática en el distrito de Ahanta Oeste podría deberse a las elevadas tasas de picaduras de *An. gambiae* y la prevalencia inicial de la infección, así como en las altas densidades de las especies vectoriales *An. melas* y *Mansonia*, que no tienen dientes o tienen muy pocos y se muestran con poca frecuencia.

Translated from English version into Spanish by Ribcamar and Natasha Ocampo, through

TRANSLATORS
WITHOUT BORDERS
